# Surgical site infection following cesarean section and its predictors in Ethiopia: A systematic review and meta-analysis

**DOI:** 10.1371/journal.pone.0296767

**Published:** 2024-03-21

**Authors:** Eyob Shitie Lake, Abebaw Alamrew, Wagaye Shumete Belay, Gizachew Yilak, Besfat Berihun Erega, Zinie Abita, Mulat Ayele

**Affiliations:** 1 School of Midwifery, College of Medicine and Health Science, Woldia University, Woldia, Ethiopia; 2 Department of Nursing, College of Medicine and Health Science, Woldia University, Woldia, Ethiopia; 3 School of Midwifery, College of Medicine and Health Science, Debre Tabor University, Debre Tabor, Ethiopia; 4 School of Public Health, College of Medicine and Health Science, Mizan Tepi University, Mizan Aman, Ethiopia; Hospital Femina, BRAZIL

## Abstract

**Introduction:**

Worldwide, surgery related deaths within 30 days of the procedure accounts the third contributor among all causes of deaths, with an estimated 4.2 million people annually and half of these deaths occur in low and middle income countries.

**Objective:**

To determine the pooled prevalence of surgical site infection following cesarean section and its predictors in Ethiopia.

**Methods:**

A systematic review and meta-analysis were conducted by using PRISMA guideline. An appropriate and comprehensive search of PubMed, MEDLINE, EMBASE, CINAHL, Google Scholar, HINARI and Scopus was done. This SRMA included all articles conducted in all regional state of Ethiopia reporting the prevalence/proportion/incidence of SSI after cesarean section and/or associated factors. All observational study designs were included in this SRMA. Articles which lack our outcome of interest: SSI following cesarean section and its predictors were excluded from this SRMA. The I^2^ statistic was used to quantify heterogeneity across studies. Funnel plot asymmetry and Egger’s tests were used to check for publication bias. A random effect model was used to estimate the pooled prevalence of SSI. Adjusted Odds Ratio (OR) with 95% Confidence Interval (CI) was also considered to determine the association of identified variables with SSI. Statistical analysis was conducted using STATA version 17 software.

**Result:**

Initially 6334 studies were identified and finally 19 studies were found eligible for the analysis. Studies with a score of 7 and above were included for the final systematic review and meta-analysis. The review was comprised of 14 cross sectional studies, 4 cohort and one case control studies. The pooled estimate of SSI in Ethiopia was 11.13% (95%CI, 9.29–12.97%). Prolonged labor (AOR = 3.16, 95% CI; (2.14–4.68)), chorioamnionitis (AOR = 4.26, 95% CI; (1.99–8.91)), prolonged PROM (AOR = 3.80, 95% CI; (2.51–5.62)), repeated vaginal examination (AOR = 3.80, 95% CI; (2.45–5.88)), decreased hemoglobin level (AOR = 4.57, 95%CI; (3.16–6.60)), vertical skin incision (AOR = 3.09, 95% CI; (2.04–4.67)) and general anesthesia (AOR = 1.82, 95% CI (1.21–2.75)) are significantly associated with SSI after cesarean section in Ethiopia.

**Conclusion:**

SSI after cesarean section in Ethiopia is high. Prolonged labor, chorioamnionits, prolonged PROM, repeated vaginal examination, decreased Hgb level, vertical skin incision and general anesthesia were positively associated. Thus, evidence based intra-partum care should be practiced.

## Introduction

Surgical site infection (SSI) is defined as an infection that occurs after surgery in the part of the body where the surgery took place. SSI after cesarean section can sometimes be superficial infections involving the skin and subcutaneous tissue or it may be more serious and can involve fascia, muscle, adjacent organs and uterus [1]. Globally, surgery related deaths within 30 days of the procedure accounts the third contributor among all causes of deaths, with an estimated 4.2 million people annually and half of these deaths occur in low and middle income countries [2]. The case fatality rate is higher even in the developed nations where advanced health facility is fulfilled [3]. In addition to substantial morbidity and mortality to patients who have undergone surgical procedures, SSI contributes extra burden by prolonging the duration of hospital stay and increasing costs for treatment as well as diagnostic tests, laboratory tests and imaging studies [4]. Furthermore, expenses for additional medical costs and supplies, losing salary for missed work and also long term complications makes it double burden for patients and their families.

The prevalence of SSI varies from place to place. For instance, a study conducted in Polish hospitals revealed a very low incidence, 0.5% [5]. On the other hand, a study conducted in Ireland and rural China revealed that SSI complicated about 0.63% [6] and 1.79% [7] of mothers delivered via cesarean section respectively. An incidence rate of 1.44% [8], 3.7% [9], 6.2% [10] and 8.54% [11] SSI were reported in Brazil, Israel, Estonia and Nepal respectively. On the contrary, substantial amount of SSI cases were reported in various parts of Africa. For instance, around 48% [12] of women in Tanzania delivered by cesarean section were complicated by SSI. Another study conducted in Nigeria and Rwanda showed that 16.01% [13] and 10.9% [14] of women were complicated by SSI following cesarean section respectively. Furthermore, another study conducted in china revealed that 23.3% of mothers developed surgical site infection following cesarean section [15]. The rate of SSI following cesarean section varies in different parts of Ethiopia. For instance, 12.9% rate of SSI in Mizan Tepi University hospital [16], 9.5% in Asela [17], 15.1% in Addis Ababa [18], 11.7% in Tigray [19] and 25.4% in [20]. This higher prevalence of SSI after cesarean section points out the presence of defect in quality of health care services. Despite various obstetric and socio demographic risk factors are responsible for its occurrence, it might also be due to lack of appropriate practice and effective implementation of evidence based practice towards SSI prevention techniques recommended by world health organization (WHO) and center of disease control and prevention (CDC).

Lots of risk factors have been reported by studies including; increased body weight, diabetes mellitus, immune suppressed, prolonged labor, prolonged rupture of membrane, chorioamnionitis, anemia, vertical type of skin incision, use of general anesthesia, repeated vaginal examination, excessive subcutaneous tissue, prolonged operation time and so on [1, 20–23]. Despite the fact that systematic review and meta-analysis (SRMA) on the topic has been done previously, there were varies inconsistent studies after that. There is no latest and updated study of the nationwide burden of SSI in Ethiopia, studies published after 2019 and cohort studies were not incorporated. Therefore, this systematic review and meta-analysis aimed to assess the current pooled prevalence of SSI following cesarean section and its associated factors in Ethiopia.

### Objective

To determine the pooled prevalence of SSI following cesarean section and its predictors in Ethiopia.

## Methods

### Search strategies

We have checked the PROSPERO database https://www.crd.york.ac.uk/prospero/display-record.php?) to see whether recently published or ongoing projects exist related to the topic to avoid any further unnecessary duplication: This systematic review and meta-analysis were registered in the PROSPERO database with ID no. CRD42023442583. A SRMA was conducted on SSI following cesarean section and its predictors in Ethiopia. Both published and unpublished literatures conducted on the prevalence and/or the factors associated with the development of SSI following cesarean delivery in Ethiopia were searched via two authors (ES and MA). Eligible literatures in English language only were identified. An appropriate and comprehensive search of PubMed, MEDLINE, EMBASE, Google Scholar, HINARI and Scopus was done since 2013. Furthermore, relevant articles found from grey literatures available on local shelves and institutional repositories were reviewed systematically. Medical Subject Headings (MeSH) and key terms had been developed using different Boolean operators ‘AND’ and ‘OR’. The following search terms were used: “(prevalence) OR (magnitude) OR (proportion) OR (epidemiology) OR (surgical site infection) OR (surgical site) OR (wound infection) AND (cesarean section) OR (cesarean delivery) OR (post cesarean section) AND (determinants) OR (associated factors) OR (risk factors) OR (predictors) AND (Ethiopia)”. The electronic literature search was last performed on September 23, 2023. Mendeley reference manager software was used to collect and manage the literatures as well as to avoid possible duplications.

### Eligibility criteria

#### Inclusion

This SRMA included all articles conducted in all regional state of Ethiopia reporting the prevalence/proportion/incidence of SSI after cesarean section and/or associated factors. All observational study designs (case-control and cross-sectional study designs) were included in this SRMA. Both published articles and grey literatures published in English language from January 1 2013 to September 2023.

#### Exclusion

which lack our outcome of interest: SSI following cesarean section and its predictors were excluded from this SRMA. On the other hand, literatures with poor quality as per the criteria of reviewing articles were excluded from this SRMA.

### Outcome measurement

This SRMA has comprised of two main outcomes. The primary outcome was magnitude of SSI among women underwent cesarean section, which is defined as the proportion of women after cesarean section who fulfills the CDC criteria of surgical site definition within 30 days of post-partum and it could be at least one of the following: (*A superficial incisional*, *deep incisional or deep/organ SSI)*. The secondary outcome was the determinants of SSI after cesarean section in Ethiopia.

### Data extraction

All the datasets were exported to Mendeley reference manager, and then we transferred to the Microsoft Excel spreadsheet to remove duplicate data in the review. Two authors (ES and MA) independently extracted all the important data using standardized data extraction format developed according to 2014 Joanna Briggs Institute (JBI) Reviewers’ manual [24]. Any disagreement between reviewers was resolved by the third author (AA). Consensus was declared through critical discussion and evaluation of the articles by all the independent reviewers. The name of the author, sample size, publication year, study region, study design, prevalence/proportion of surgical site infection, and adjusted odds ratio with its 95% CI of the factors associated with SSI. Articles which fulfilled the predetermined criteria were used as a source of data for the final analysis.

### Quality assessment

Once the database results exported to mendeley reference manager and duplicate results removed, we used Newcastle–Ottawa Quality Assessment scale (NOS) adapted for observational studies to assess the quality of each study included in this SRMA [25] (S1 Table). This quality assessment scale evaluates the literatures in three categories:

- Selection (4 points)
- Comparability(2points) and
- Outcome (3 points)

Two Authors (ES and MA) assessed the quality of each study (i.e. methodological quality, sample selection, sample size, comparability and the outcome, and statistical analysis of the study). In the case of disagreement between two authors; another third author (AA, WS and BB) were involved and discussed and resolved the disagreement.

### Data processing and analysis

The extracted Microsoft Excel spreadsheet format data was imported to STATA software version 17 (STATA corporation, Texas, USA) for analysis. Then random effect model was used to estimate the pooled prevalence of SSI after cesarean section in Ethiopia. Cochrane Q-test and I^2^ statistics were computed to assess heterogeneity among all the studies included in this SRMA. Accordingly, if the result of I ^2^ is 0–40% it is mild heterogeneity, 40 to 70% would be moderate heterogeneity, and 70 to 100% would be considerable heterogeneity [26]. Funnel plot and Eggers test were done to assess publication bias. The p-value>0.05 indicated that there was no publication bias. Subgroup analysis was done based on the study region. A forest plot format was used to present the pooled prevalence of surgical site infection after cesarean section with 95% CI. To identify determinant factors, we used the pooled AOR in forest plot format with its respective 95%CI.

### Subgroup and sensitivity analyses

Based on the study region, subgroup analyses were done. The stability or robustness of the pooled estimates to outliers and the impact of individual studies were assessed using sensitivity analysis.

## Result

**Fig 1:**
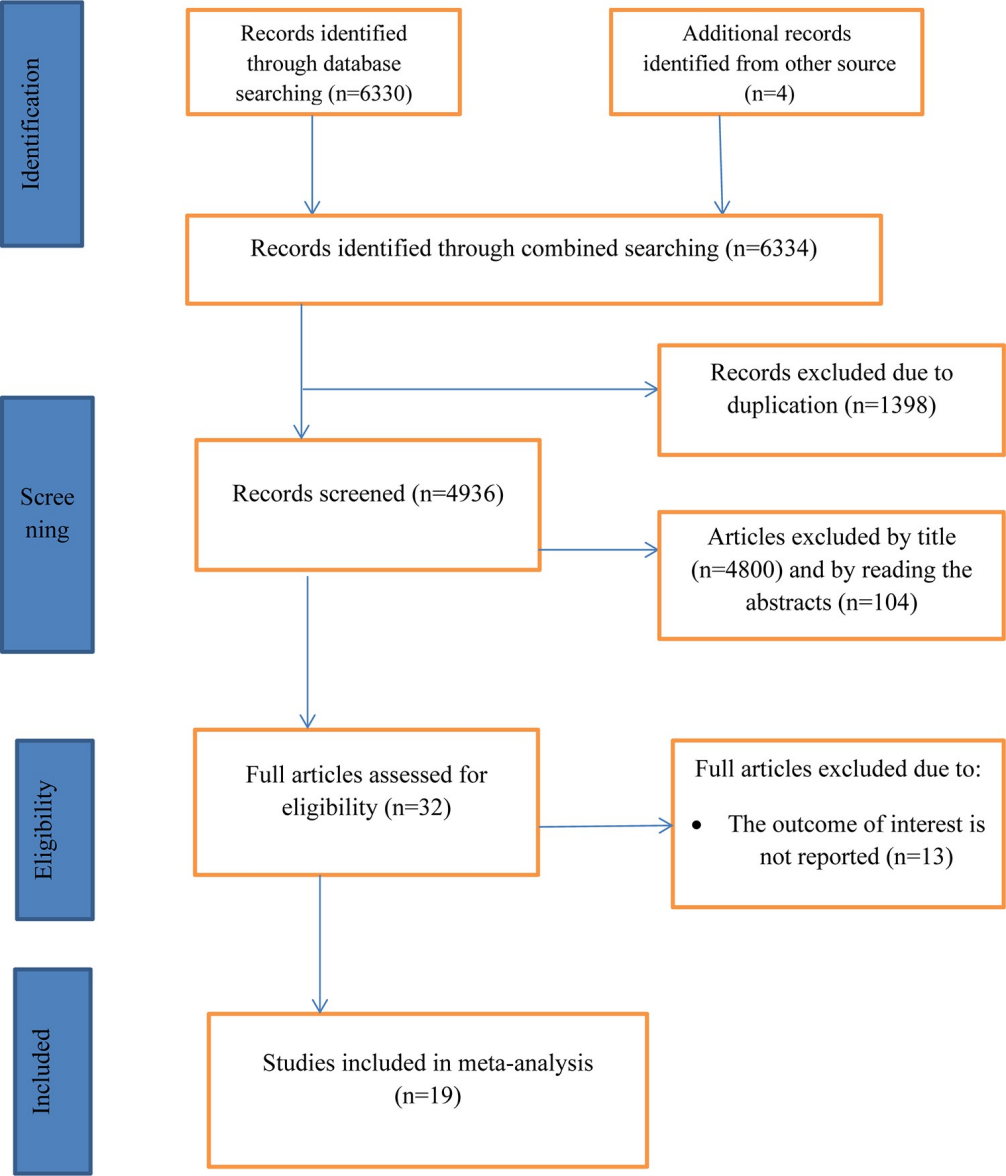
PRISMA flow chart for the selection of systematic review of surgical site infection after cesarean section and its associated factors in Ethiopia.

A total of 6334 articles were found by using our search strategies: Google scholar, pub-med, Hinari, EMBASE, Scopus and Medline. After 1398 articles were removed for the reason of duplication, 4932 articles left. Then by reviewing their titles and abstracts, 4800 and 100 articles were removed respectively. Finally 32 full text papers were accessed and evaluated for the predefined inclusion criteria. Thus 13 more articles were excluded for the afore-mentioned reasons. Eventually, 19 articles were found eligible for inclusion in the final SRMA (Fig 1).

Among the studies included in this SRMA, six studies were in Amhara, four in SNNP region, two in Oromia, two in Addis Ababa, two in Harari, one in Dire Dawa and two in Tigray. Fourteen of the studies were cross sectional, four were cohort and one case control studies. A total of 8755 participants with the smallest 166 to the largest 1069 participants included and found the prevalence which ranges from 6.80 to 25.4% of SSI following cesarean section in Ethiopia. On the other hand, the quality of each study was assessed by using NOS, the score of all included studies lies between 8 to 9 which indicates good quality (Table 1) (1).

**Table 1:** Characteristics of included studies in the SRMA on SSI after cesarean section in Ethiopia.

Seri no	Author	Year	Region	Study design	Sample size	Prevalence of SSI (%)	Quality
1	Gedefaw G et al [27].	2018	Amhara	Cross sectional	447	9.4	Good
2	Molla M et al [28].	2019	Amhara	Cross sectional	347	8.1	Good
3	Bizuayew H et al [22].	2021	Amhara	Cross sectional	633	12.4	Good
4	Ali O et al [29].	2022	Amhara	Cross sectional	818	12.2	Good
5	Azeze G and Bizuneh A [30].	2019	Amhara	Cross sectional	383	7.8	Good
6	Gashaw A et al [31].	2022	SNNP	Cross sectional	431	11.8	Good
7	Wodajo S et al [23].	2017	SNNP	Cross sectional	600	11	Good
8	Anjelo et al [32].	2018	SNNP	Cross sectional	325	12.9	Good
9	Ayala D et al [33].	2021	Oromia	Cross sectional	401	8.9	Good
10	Mamo T et al [17].	2017	Oromia	Cross sectional	384	9.5	Good
11	Worku M and Abdela A [34].	2018	Addis Ababa	Cross sectional	474	8.4	Good
12	Alemye T et al. [35].	2021	Harari	Cross sectional	1069	12.3	Good
13	Wendmagegn T et al. [19].	2018	Tigray	Cross sectional	206	11.7	Good
14	Gelaw K et al. [21].	2017	Tigray	Cross sectional	384	6.8	Good
15	Ketema D et al [36].	2020	Amhara	Cohort	520	25.4	Good
16	Wae M et al [37].	2017	SNNP	Cohort	488	12	Good
17	Lijaemiro H et at [18].	2020	Addis Ababa	Cohort	166	15.1	Good
18	Adane A et al [20].	2023	Harari	Cohort	337	7.4	Good
19	Dessu S et al [38].	2021	Dire Dawa	Case control	476		Good

### Magnitude of SSI after cesarean section in Ethiopia

**Fig 2:**
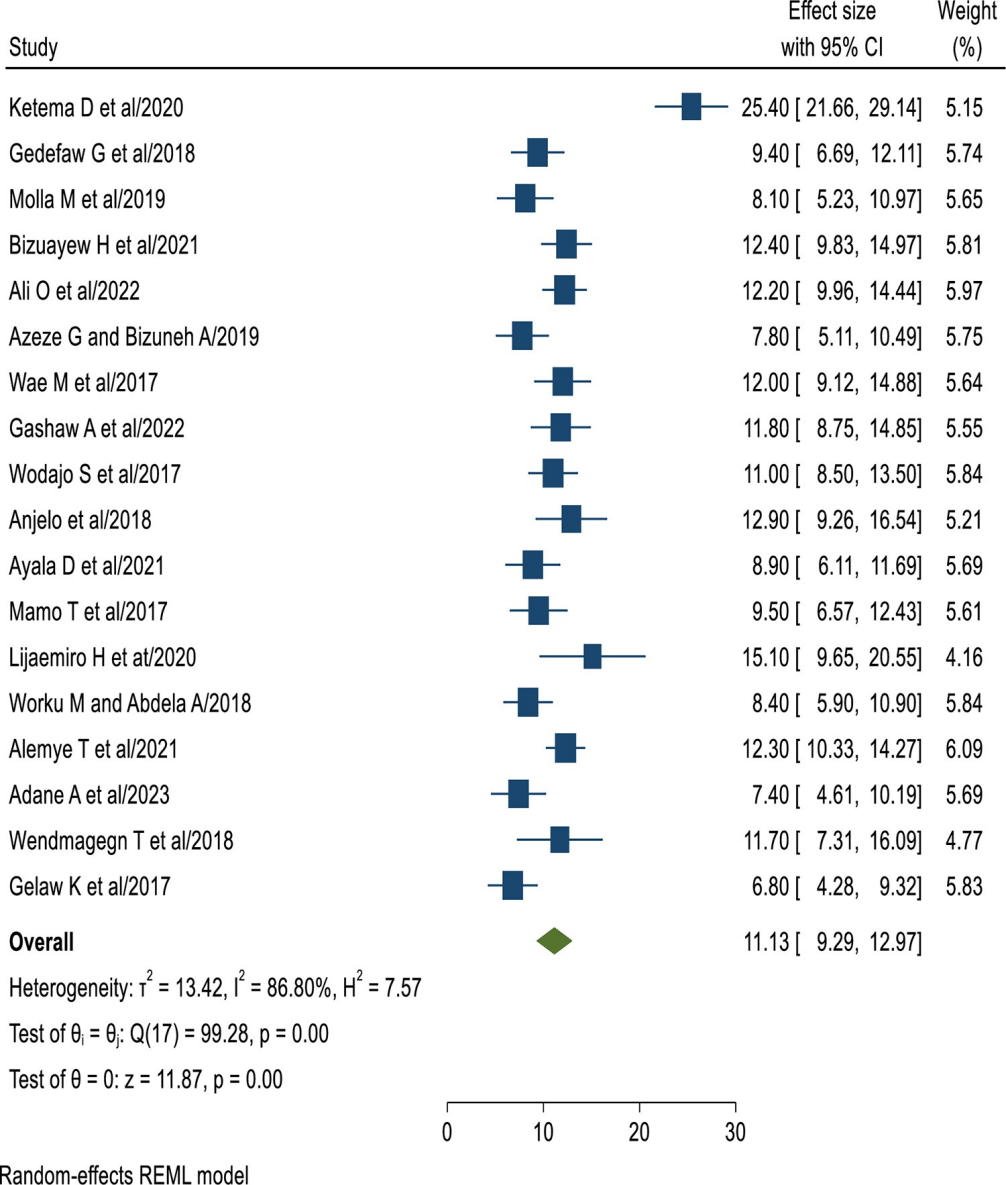
The pooled prevalence of SSI after cesarean section in Ethiopia, 2023.

The finding of this SRMA showed that the pooled prevalence of surgical site infection after cesarean section in Ethiopia was 11.13% (95% CI: 9.29, 12.97%), and there is significant heterogeneity between the studies as evidenced by the Cochrane heterogeneity index (I^2^ = 86.80%), P = 0.00. Therefore we have used the random effect model to resolve the issue of heterogeneity between included studies. Moreover, we have considered subgroup analysis as a potential way of addressing heterogeneity. The finding was presented using a forest plot (Fig 2).

### Publication bias

**Fig 3:**
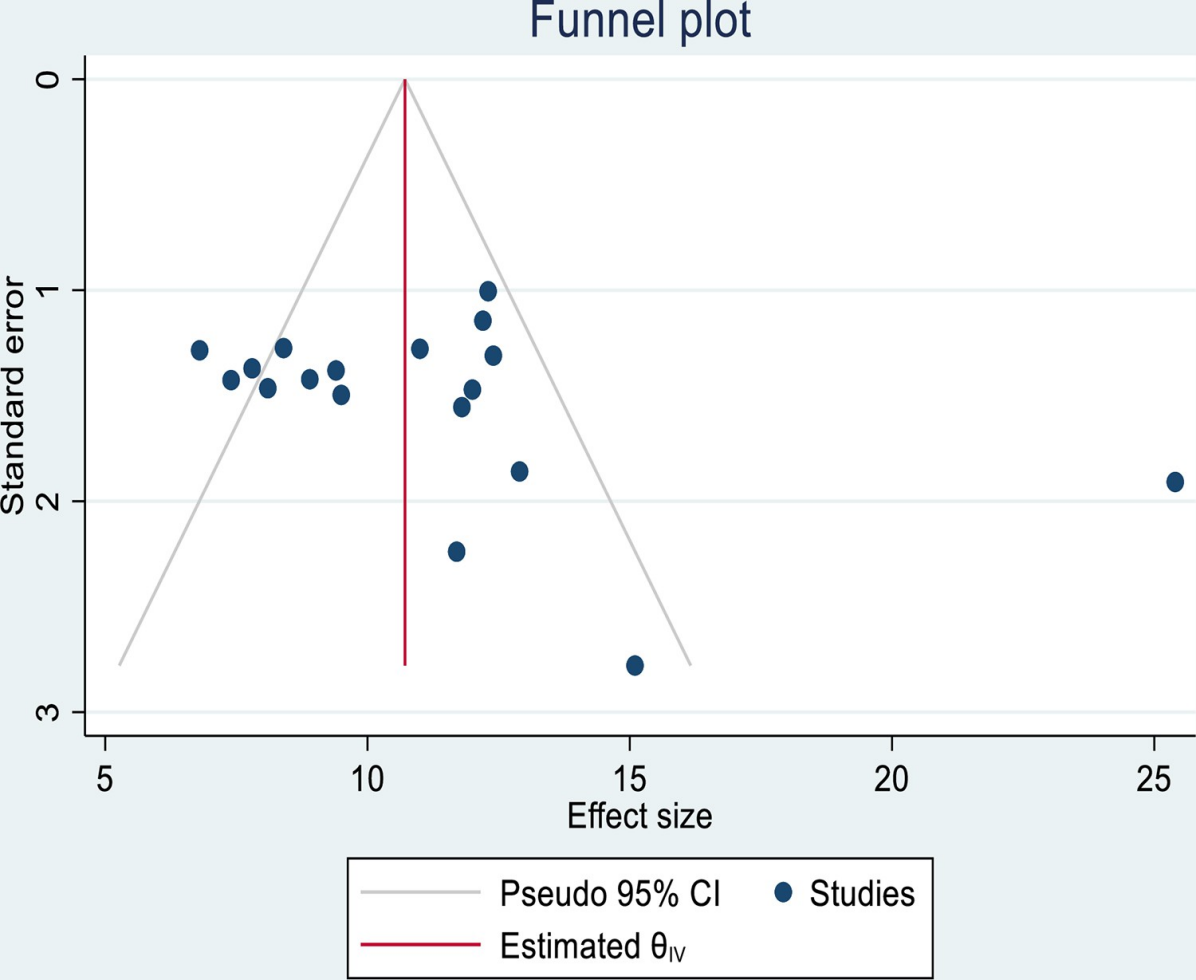
Funnel plot showing the symmetric distribution of articles on SSI after cesarean section in Ethiopia, 2023.

The presence or absence of publication bias was verified by using statistical analysis (funnel plot and egger’s test (P = 0.0502 (P >0.05)) result showed no publication bias. However, the substantial heterogeneity between included studies might affect the overall pooled prevalence of SSI after cesarean section (Fig 3). Thus subgroup analysis was done.

### Subgroup analysis of SSI following cesarean section in Ethiopia

**Fig 4:**
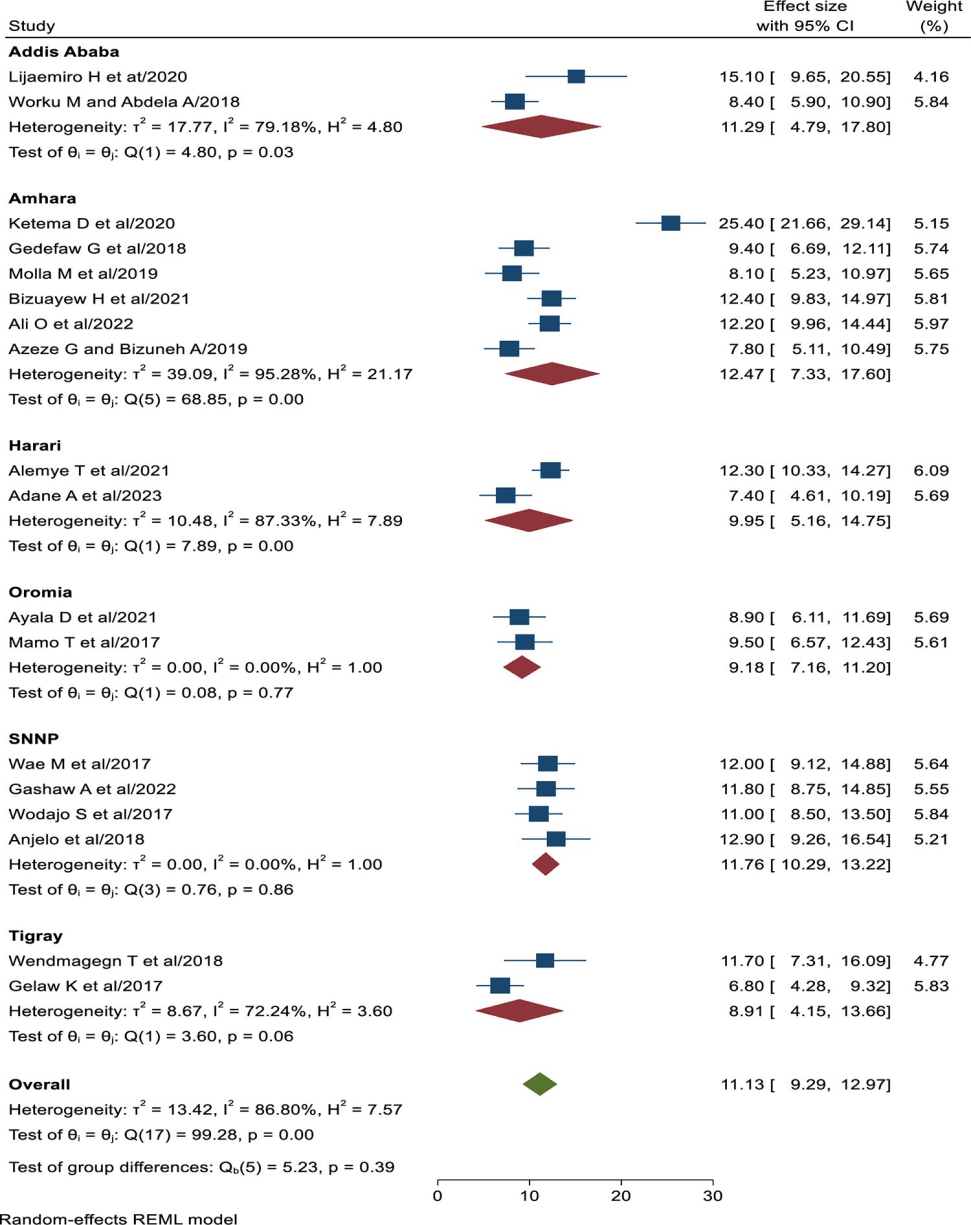
Forest plot showing sub group analysis of SSI after cesarean section by the study region in Ethiopia, 2023.

Subgroup analysis was done by using the study region and the result revealed that the pooled prevalence of SSI cesarean section was highest in Amhara region (12.47%; 95% (CI: 7.33, 17.60%), I^2^ = 95.28%, P = 0.00) followed by SNNP region (11.76%; 95% (CI: 10.29, 13.22%), I^2^ = 0.00%, P = 0.86). on the contrary, the lowest prevalence of SSI after cesarean section was observed in Tigray region (8.91%; 95%CI: 4.15, 13.66%), I^2^ = 72.24%, P = 0.06) followed by Oromia region (9.18%; 95%CI: 7.16, 11.20%), I^2^ = 0.00%, P = 0.77 (Fig 4).

### Sensitivity analysis

**Fig 5:**
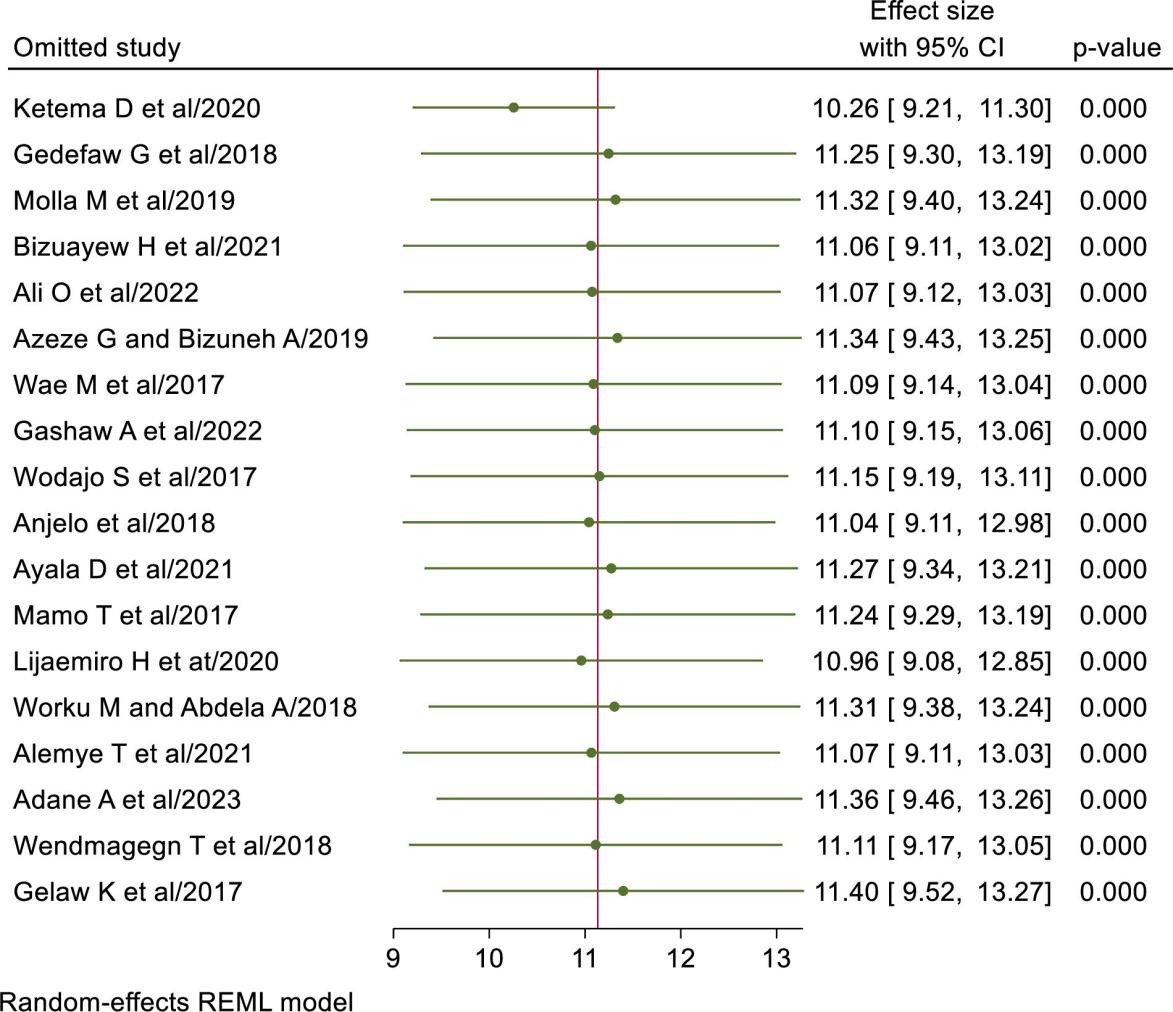
Sensitivity analysis of SSI after cesarean section in Ethiopia, 2023.

The result of a random effect model revealed that, the pooled prevalence of SSI after cesarean section in Ethiopia was not influenced by a single study (Fig 5).

### Determinants of SSI following cesarean section in Ethiopia

In this systematic review and meta-analysis; seven factors that are associated with surgical site infection following cesarean section at least in three primary studies were included in the identification of the predictors of surgical site infection after cesarean section. Accordingly, prolonged labor, repeated digital vaginal examination, prolonged rupture of membrane, chorioamnionitis, decreased post-operative hemoglobin level, vertical skin incision and general anesthesia were significantly associated with surgical site infection after cesarean section in Ethiopia (Table 2). Mothers who had prolonged labor before delivery were 3.16 times more likely to develop surgical site infection after cesarean section than their counterparts (AOR = 3.16, 95% CI; (2.14–4.68)). The relationship between surgical site infection and chorioamnionitis were also tested, that women with chorioamnionitis were 4.26 times more likely to have surgical site infection post cesarean section than those who had no (AOR = 4.26, 95% CI; (1.99–8.91)). The likelihood of developing surgical site infection among mothers who had prolonged rupture of membrane was 3.80 times more than those who had no prolonged rupture of membrane (AOR = 3.80, 95% CI; (2.51–5.62)). Repeated digital vaginal examination increased the risk of surgical site infection by 3.80 times than the counter parts (AOR = 3.80, 95% CI; (2.45–5.88)). Mothers with decreased post-operative hemoglobin level after cesarean section were 4.57 times more likely to develop surgical site infection than those with normal hemoglobin level (AOR = 4.57, 95%CI; (3.16–6.60)). Women with vertical type of skin incision for cesarean section were 3.09 times more likely of developing surgical site infection than mothers with supra pubic transverse skin incision (AOR = 3.09, 95% CI; (2.04–4.67)). Mothers whose cesarean section was done by general anesthesia were 1.82 times at increased risk developing surgical site infection than mothers who were provided with spinal anesthesia (AOR = 1.82, 95% CI (1.21–2.75)).

**Table 2:** Factors associated with SSI after cesarean section in Ethiopia.

Variables	Author/ year of publication	AOR	95% CI	Pooled AOR	95% CI
**Prolonged labor**	Gedefaw G et al/2018	5.25	2.32–11.8	**3.16**	2.14–4.68
	Bizuayew H et al/2021	3.48	1.49–8.08		
	Ali O et al/2022	1.26	0.62–2.58		
	Wae M et al/2017	1.44	1.04–2.47		
	Gashaw A et al/2022	4.05	1.12–13.71		
	Wodajo S et al/2017	6.78	2.54–18		
	Ayala D et al/2021	4.12	1.01–32.19		
	Worku M and Abdela A /2018	3.57	1.92–9.42		
	Adane A et al/2023	4.04	1.52–10.79		
	Wendmagegn T et al/2018	6.06	1.67–21.95		
	Gelaw K et al/2017	3.48	1.25–9.68		
**Chorioamnionitis**	Gedefaw G et al/2018	3.73	1.22–11.4.	**4.26**	1.99–8.91
	Molla M et al/2019	4.37	1.53–12.5		
	Ali O et al/2022	6.46	1.82–22.7		
	Mamo T et al/2017	9.05	2.35–34.83		
	Alemye T et al/2021	1.02	0.64–1.61		
	Dessu S et al/2021	5	1.05–23.9		
	Wendmagegn T et al/2018	16.17	2.85–91.8		
**Repeated PV examination**	Ketema D et al/2020	1.88	1.71–3.2	**3.80**	2.45–5.88
	Azeze G and Bizuneh A/2019	2.47	0.46–13.13		
	Gashaw A et al/2022	6.1	2.15–17.35		
	Wodajo S et al/2017	8.59	1.74–42.23		
	Ayala D et al/2021	0.53	0.05–4.82		
	Lijaemiro H et at/2020	13.07	1.02–168.1		
	Worku M and Abdela A/2018	5.24	8.46–16.2		
	Adane A et al/2023	4.77	1.87–12.74		
	Dessu S et al/2021	4.2	2.16–8.22		
**Post op HGB**	Molla M et al/2019	5.28	1.97–14.18	**4.57**	3.16–6.60
	Bizuayew H et al/2021	3.221	1.25–8.31		
	Ali O et al/2022	3.44	1.56–7.56		
	Gashaw A et al/2022	3.16	1.2–8.38		
	Wodajo S et al/2017	2.62	1.21–5.69		
	Anjelo et al/2018	0.86	0.011–1.68		
	Ayala D et al/2021	4.51	1.84–11.07		
	Mamo T et al/2017	7.78	2.37–25.54		
	Worku M and Abdela A/2018	16.34	12.98–30.4		
	Alemye T et al/2021	4.1	2.61–6.44		
	Adane A et al/2023	3.42	1.32–8.87		
	Dessu S et al/2021	6.9	3.45–14.1		
	Gelaw K et al/2017	2.43	0.67–8.85		
**Prolonged PROM**	Ketema D et al/2020	1.5	1.31–1.64	**3.80**	2.51–5.62
	Gedefaw G et al/2018	1.98	0.76–5.1		
	Bizuayew H et al/2021	4.61	2.34–9.09		
	Ali O et al/2022	2.94	1.52–5.67		
	Gashaw A et al/2022	4.12	1.5–11.27		
	Wodajo S et al/2017	5.83	2.14–15.89		
	Mamo T et al/2017	3.77	1.53.9.31		
	Lijaemiro H et at/2020	3.98	0.38–41.61		
	Worku M and Abdela A/2018	7.84	4.25–12.34		
	Dessu S et al/2021	5.4	1.84–15.87		
	Wendmagegn T et al/2018	8.82	2.17–35.82		
**Vertical skin incision**	Ketema D et al/2020	2.6	1.05–6.44	**3.09**	2.04–4.67
	Gedefaw G et al/2018	1.6	0.67–3.74		
	Molla M et al/2019	5.19	1.87–14.37		
	Azeze G and Bizuneh A/2019	4.77	1.74–13.08		
	Ayala D et al/2021	1.27	0.26–6.01		
	Lijaemiro H et at/2020	0.046	0.001–2.41		
	Adane A et al/2023	3.76	1.47–9.58		
	Gelaw K et al/2017	5.73	2.05–16.0		
**General Anesthesia**	Ketema D et al/2020	0.69	0.29–1.6	**1.82**	1.21–2.75
	Gedefaw G et al/2018	1.34	0.35–5.1		
	Wae M et al/2017	2.45	1.38–4.35		
	Ayala D et al/2021	3.96	1.02–15.29		
	Alemye T et al/2021	2.02	1.34–3.02		
	Gelaw K et al/2017	2.13	0.66–6.83		

## Discussion

SSI is the most common health care associated infections in developing nations. However, integrating the range of preventive measures before, during and after the surgery is very compulsory to prevent its occurrence [39]. Despite various methods of infection prevention techniques have been proposed and recommended by WHO, its prevalence has been continued substantially. The main aim of conducting this SRMA was to identify the pooled prevalence of SSI following and its predictors in Ethiopia. The result of this SRMA showed the overall pooled prevalence of SSI following cesarean section in Ethiopia was 11.13% with a 95% CI: (9.29, 12.97%), which is much higher than the global prevalence of 5.63% [40]. This result is consistent with a study conducted in Rwanda which is 10.9% [14]. However, this was lower than the study conducted in China [15], Jordan [41] and Ekiti state, Nigeria [13] reported 23.3%, 14.4% and 16.01% respectively. The reason for this discrepancy might be due to the smaller sample size used by the above studies (most of the studies conducted outside Ethiopia were observational study designs and small sample size recruited). Furthermore, the institution level of which the study conducted in China was tertiary hospitals. Thus, referral cases are to be found there more likely than other primary level of institutions. On the contrary, our study finding was much higher than the studies conducted in developed nations: Ireland [42], Poland [5], Estonia [10] and Egypt [43] reported 0.63%, 0.5%, 6.2% and 5.34% respectively. The inconsistency from these studies might be due to the difference in health care facility, thus evidence based practice and effective infection prevention mechanisms are more likely to be practiced in developed countries. The operation theatre setup in developed societies is established at the minimum standards of patient safety. The finding of this study implies that the optimum level of cesarean section should be done in tandem with the initiatives to reduce post-surgical site infection.

This review has also identified the factors associated with SSI following cesarean section in Ethiopia. Prolonged labor, chorioamnionitis, prolonged PROM, vertical skin incision, decreased post- operative HGB, repeated vaginal examination and general anesthesia were found to be the independent risk factors of surgical site infection following cesarean section in Ethiopia. Women who had prolonged labor were 3.16 times more likely to develop SSI than their counterparts. The finding was similar with the studies conducted in Cross river state, Nigeria [44] and Egypt [43]. This is explained as the nature of labor pain causes maternal dehydration and fatigue especially when the duration of labor becomes longer. On another way, the longer the duration of labor, the more repeated vaginal examination to be done. This expedites ascending infection to occur and eventually SSI develops.

Mothers after cesarean section who had chorioanionitis during labor and delivery were about 4.26 times more likely to develop SSI than their counter parts. This study finding was similar with the studies conducted in Estonia [10], Poland [5] and Egypt [43]. This is due to the infection involving the intra amniotic area, which is due to ascending migration of the normal floras (microorganisms in cervico-vaginal area) towards the uterine through the endo-cervical canal. The further migration of these microbes to the surgical site during cesarean section can easily access the maternal tissues breached by the operation.

On the other hand, the association between SSI following cesarean section and prolonged PROM was tested. The likelihood of developing SSI after cesarean section was nearly four times higher than their counterparts. The finding of this review was consistent with other studies conducted in calabar, Nigeria [44] and Nepal [11]. The presence of feto-placental membranes prevents ascending infections which eventually causes intra amniotic infections. The rupture of this important barrier for longer duration apparently increases the risk of infection around the surgical area.

Hemoglobin level below 11 gm/dl post cesarean section was found as a predictor of SSI. Women with decreased hemoglobin level were 4.57 times more likely to have SSI after cesarean section than those with normal hemoglobin level. This finding is supported with studies conducted in Poland [5], China [15] and Egypt [43]. Decreased iron level in the mother’s blood circulation obliterates the capacity of infection prevention. On the other hand, the lower hemoglobin level causes disrupted vasculature and deprived oxygenation to the maternal tissues. This deprived oxygenation delays the wound healing process after the operation and this eventually leads to post procedure infection [45]

Repeated digital vaginal examination during labor is found to be independently associated with SSI following cesarean section. The likelihood of developing surgical site infection is 3.8 timely higher than their counterparts. This is consistent with studies conducted in Nepal [11]. The possibility of contamination from vagina to the endometrium and ultimately to the uterine wall increases as the number of digital vaginal examination increases, which will eventually crosses to the incision site during cesarean section and consequently SSI arises.

The type of skin incision for cesarean section had also a risk of SSI, with a likelihood of 3.09 times higher in the case of vertical skin incision than their counterparts. This study finding is supported by a study conducted in Nigeria [46] and Nepal [47]. This is due to vertical type of skin incision during cesarean section is associated with decreased tensile strength of the wound and delayed healing process. Thus, mothers post procedures are prone to wound dehiscence and eventually puts them at risk of post procedure surgical site infection [48]. the other possible explanation is usually vertical skin incision for cesarean section is used for complicated obstetrical emergencies like in obstructed labor, uterine rupture and chorioamnionitis. Thus, the aforementioned obstetrical emergencies by themselves are highly suggestive of maternal sepsis. Furthermore, vertical skin incision is done in the area of less vascular when compared to the Pfannenstiel skin incision, which delays the wound healing.

The odds of developing SSI after cesarean section among women with general anesthesia were about 1.82 times more likely than spinal anesthesia. This study finding was supported by similar studies conducted in China [49]. This is explained as some inhalational anesthesia produces dose dependent uterine relaxation, which causes uterine atony and hemorrhage. Ultimately the deprived oxygenation of body tissues results in delayed wound healing and SSI [45]. On the other hand, women after general anesthesia requires longer time period to get fully recovered from the anesthesia than regional anesthesia. This results in delayed ambulation of women post procedure, which is very crucial in wound healing process.

## Conclusion

The result of this SRMA showed that the prevalence of surgical site infection after cesarean section is high in Ethiopia, which indicates the need of focused and meticulously planned interventions should be designed and implemented to decrease the prevalence of surgical site infection following cesarean section so as to decrease the tragedies of maternal morbidity and mortalities. Determinant factors OF SSI after cesarean section in Ethiopia were prolonged duration of labor, chorioamnionitis, prolonged PROM, repeated vaginal examinations, decreased hemoglobin level, vertical skin incision and general anesthesia. Therefore, evidence based practice before, during and after labor and delivery should be practiced to avoid prolonged duration of labor, chorioamnionitis and repeated vaginal examination. Furthermore, patients with low hemoglobin level and prolonged PROM should be exhaustively treated to reduce the risk of SSI. Complicated intra-partum cases are more likely to have vertical type of skin incision during cesarean section and general anesthesia might be needed to do so, thus special attention should be given prevent complicated labor such as obstructed labor, chorioamnionitis and uterine rupture, which eventually reduces the risk of women from developing surgical site infection.

## Supporting information

S1 Checklist PRISMA 2020 checklist. (DOCX)

S1 Table Newcastle-Ottawa quality assessment scale used for this SRMA. (DOCX)

## Abbreviations

CDC: Center of Disease Control and Prevention
NOS: Newcastle Otawa Quality Assessment Scale
SSI: Surgical Site Infection
SRMA: Systematic Review and Meta Analysis
PRISMA: Preferred Reporting Items for Systematic Review and Meta-Analysis
SNNPR: South Nation Nationalities and People Region
WHO: World Health Organization
