# Supplementary material for: Surgical site infection following cesarean section and its predictors in Ethiopia: A systematic review and meta-analysis
Source: PLoS One. 2024 Mar 21;19(3):e0296767. doi: 10.1371/journal.pone.0296767 (PMC10956825; doi:10.1371/journal.pone.0296767)
Supplement: S1 Table — (DOCX) [file pone.0296767.s002.docx]

Supplementary file 1: Newcastle-Ottawa Quality Assessment Scale for cross sectional studies used in the systematic review and meta-analysis, surgical site infection after caesarean section and its predictors in Ethiopia 2023

| \|  \| **Selection (4)** \| \| \| \| **Comparability(2)** \| **Outcome (3)** \| \| **Total score** \| \| --- \| --- \| --- \| --- \| --- \| --- \| --- \| --- \| --- \| \| Author name \| Representativeness(1) \| Sample size(1) \| Non respondents (1) \| Ascertainment of the exposure risk factor (1) \| The subjects in different outcome groups are comparable, based on the study design or analysis. confounding factors are controlled (2) \| Assessment of the outcome (2) \| Statistical test (1) \|  \| \| Gedefaw G et al. (25) \| 1 \| 1 \| 1 \| 1 \| 1 \| 2 \| 1 \| **8** \| \| Molla M et al. (26) \| 1 \| 1 \| 1 \| 1 \| 2 \| 2 \| 1 \| **9** \| \| Bizuayew H et al. (19) \| 1 \| 1 \| 1 \| 1 \| 1 \| 2 \| 1 \| **8** \| \| Ali O et al. (27) \| 1 \| 1 \| 1 \| 1 \| 2 \| 2 \| 1 \| **9** \| \| Azeze G and Bizuneh A. (28) \| 1 \| 1 \| 1 \| 1 \| 2 \| 2 \| 1 \| **9** \| \| Gashaw A et al. (29) \| 1 \| 0 \| 1 \| 1 \| 2 \| 2 \| 1 \| **8** \| \| Wodajo S et al. (20) \| 1 \| 1 \| 1 \| 1 \| 1 \| 2 \| 1 \| **8** \| \| Anjelo et al. (30) \| 1 \| 1 \| 1 \| 1 \| 1 \| 2 \| 1 \| **8** \| \| Ayala D et al. (31) \| 1 \| 1 \| 1 \| 0 \| 2 \| 2 \| 1 \| **8** \| \| Mamo T et al. (32) \| 1 \| 1 \| 1 \| 1 \| 1 \| 2 \| 1 \| **8** \| \| Worku M and Abdela A. \| 1 \| 1 \| 1 \| 1 \| 2 \| 2 \| 1 \| **9** \| \| Alemye T et al. (33) \| 1 \| 1 \| 1 \| 1 \| 2 \| 2 \| 1 \| **9** \| \| Wendmagegn T et al. (34) \| 1 \| 1 \| 1 \| 1 \| 2 \| 2 \| 1 \| **9** \| \| Gelaw K et al. (18) \| 1 \| 1 \| 1 \| 1 \| 2 \| 2 \| 1 \| **9** \| \| Ketema D et al(35). \| 1 \| 1 \| 1 \| 1 \| 2 \| 2 \| 1 \| **9** \| \| Wae M et al(36). \| 1 \| 1 \| 1 \| 1 \| 2 \| 2 \| 0 \| **8** \| \| Lijaemiro H et at(37). \| 1 \| 0 \| 1 \| 1 \| 2 \| 2 \| 1 \| **8** \| \| Adane A et al(17). \| 1 \| 1 \| 1 \| 1 \| 2 \| 2 \| 1 \| **9** \| \| Dessu S et al(38) \| 1 \| 1 \| 1 \| 1 \| 2 \| 1 \| 1 \| **8** \| |
| --- | --- | --- | --- | --- | --- | --- | --- | --- | --- | --- | --- | --- | --- | --- | --- | --- | --- | --- | --- | --- | --- | --- | --- | --- | --- | --- | --- | --- | --- | --- | --- | --- | --- | --- | --- | --- | --- | --- | --- | --- | --- | --- | --- | --- | --- | --- | --- | --- | --- | --- | --- | --- | --- | --- | --- | --- | --- | --- | --- | --- | --- | --- | --- | --- | --- | --- | --- | --- | --- | --- | --- | --- | --- | --- | --- | --- | --- | --- | --- | --- | --- | --- | --- | --- | --- | --- | --- | --- | --- | --- | --- | --- | --- | --- | --- | --- | --- | --- | --- | --- | --- | --- | --- | --- | --- | --- | --- | --- | --- | --- | --- | --- | --- | --- | --- | --- | --- | --- | --- | --- | --- | --- | --- | --- | --- | --- | --- | --- | --- | --- | --- | --- | --- | --- | --- | --- | --- | --- | --- | --- | --- | --- | --- | --- | --- | --- | --- | --- | --- | --- | --- | --- | --- | --- | --- | --- | --- | --- | --- | --- | --- | --- | --- | --- | --- | --- | --- | --- | --- | --- | --- | --- | --- | --- | --- | --- | --- | --- | --- | --- | --- | --- | --- | --- | --- | --- | --- | --- | --- |
